# Neuromyelitis Optica Spectrum Disorder in Central America and the Caribbean: A Multinational Clinical Characterization Study

**DOI:** 10.3390/neurolint14010023

**Published:** 2022-03-17

**Authors:** Fernando Gracia, Deyanira Ramírez, Alexander Parajeles-Vindas, Alejandro Díaz, Amado Díaz de la Fé, Nicia Eunice Ramírez Sánchez, Romy Castro Escobar, Luis Alberto García Valle, Roberto Weiser, Biany Santos, Awilda Candelario, Aron Benzadon, Pahola Araujo, Carlos Valderrama, Mario Larreategui, Gabriela Carrillo, Karla Gracia, Johana Vázquez-Céspedes, Priscilla Monterrey-Alvarez, Kenneth Carazo-Céspedes, Alfredo Sanabria-Castro, Gustavo Miranda-Loria, Andrea Balmaceda-Meza, Ligia Ibeth Portillo Rivera, Irma Olivera Leal, Luis Cesar Rodriguez Salinas, Arnold Thompson, Ericka López Torres, Daniel Enrique Pereira, Carolina Zepeda, César Abdón López, Ernesto Arturo Cornejo Valse, Karla Zinica Corea Urbina, Marco Antonio Urrutia, Ivonne Van Sijtveld, Blas Armien, Victor M. Rivera

**Affiliations:** 1 Neurology Service, Hospital Santo Tomás, Panama City 0819-03752, Panama; gcarrillopujolmd@gmail.com; 2 School of Medicine, Universidad Interamericana de Panamá, Panama City 0830-00929, Panama; kgraciapbg@gmail.com; 3 Neurology Service, Hospital Docente Padre Billini, Santo Domingo City 11102, Dominican Republic; dradeyaramirez@hotmail.com (D.R.); awildacandelario@hotmail.com (A.C.); 4 Neurology Service, Hospital San Juan de Dios CCSS, San Jose 1475-1000, Costa Rica; aparajeles@yahoo.com (A.P.-V.); jmvc84@hotmail.com (J.V.-C.); carazo.ces@gmail.com (K.C.-C.); asccheo@yahoo.com (A.S.-C.); 5 Neurology Service, Instituto Guatemalteco de Seguridad Social, Guatemala City 1010, Guatemala; ajdj1978@gmail.com; 6 Neurology Service, Centro Internacional de Restauración Neurológica (CIREN), La Habana 11300, Cuba; amado.diaz.fe@gmail.com; 7 Neurology Service, Hospital Dr. Mario Catarino Rivas, San Pedro Sula 21104, Honduras; ners71@yahoo.com (N.E.R.S.); thompsonarnold@yahoo.com (A.T.); 8 Neurology Service, Instituto Salvadoreño del Seguro Social, San Salvador 1101, El Salvador; romy_de_escobar@hotmail.com (R.C.E.); danielpereira_c@yahoo.es (D.E.P.); ernestocornejovalse@yahoo.com (E.A.C.V.); 9 Neurology Service, Hospital Militar Escuela Dr. Alejandro Dávila Bolaños, Managua 14285, Nicaragua; luisneuro2009@gmail.com (L.A.G.V.); zinicaurbina@yahoo.es (K.Z.C.U.); 10 Neurology Service, Hospital Horacio Oduber, Oranjestad 569, Aruba; rjwb33@hotmail.com (R.W.); ivansijtveld@gmail.com (I.V.S.); 11 Neurology Service, Hospital Cabral y Baez, Santiago City 10206, Dominican Republic; drabianysantos@hotmail.com; 12 Neurology Service, Complejo Hospitalario Dr. Arnulfo Arias Madrid CSS, Panama City 0831-01654, Panama; aronbenz@medicospaitilla.com (A.B.); paholaaraujo@gmail.com (P.A.); 13 Neurology Service, Hospital Regional Rafael Hernández CSS, David City 0816-06808, Panama; carlosarenilla@hotmail.com; 14 Neurology Service, Hospital Regional Anita Moreno MINSA, La Villa de Los Santos 0819-11380, Panama; mlarri@cableonda.net; 15 Neurology Service, Hospital San Rafael de Alajuela CCSS, Alajuela 1481-2100, Costa Rica; priscimonty@yahoo.com (P.M.-A.); gustavomiranda8363@gmail.com (G.M.-L.); 16 Neurology Service, Centro de Desarrollo Estratégico e Información de Salud y Seguridad Social (CENDEISSSS CCSS), San Jose 1475-1000, Costa Rica; andrea_bm07@hotmail.com; 17 Neurology Service, Hospital General de Enfermedades (IGSS), Guatemala City 1015, Guatemala; portilloibeth@gmail.com; 18 Neurology Service, Hospital Hermanos Almejeira, La Habana 10200, Cuba; oliverairma62@gmail.com; 19 Neurology Service, Instituto Hondureño de Seguridad Social, Tegucigalpa 11101, Honduras; luis_cesar3@outlook.es; 20 Neurology Service, Hospital Nacional Rosales, San Salvador 1101, El Salvador; dra.erickalopez@hotmail.com (E.L.T.); krito172006@hotmail.com (C.Z.); cesar051090@gmail.com (C.A.L.); 21 Neurology Service, Hospital Carlos Roberto Huembes—Policía Nacional, Managua 14203, Nicaragua; 22 Neurology Service, Hospital Infantil de Nicaragua Manuel de Jesús Rivera La Mascota, Managua 12001, Nicaragua; marcurrutia@hotmail.com; 23 Dirección de Investigación, Instituto Conmemorativo Gorgas de Estudios de la Salud, Panama City 0816-02593, Panama; 24 National Research System (SNI), National Secretary of Research Technology and Innovation (SENACYT), Panama City 0816-02852, Panama; 25 Neurology Department, Baylor College of Medicine, Houston, TX 77025, USA; vrivera@bcm.edu

**Keywords:** neuromyelitis optica spectrum disorder, Central America, Caribbean, anti-AQP4-IgG antibodies, anti-MOG-IgG antibodies, clinical characterization

## Abstract

Here, a study of NMOSD in Central America and the Caribbean with a multinational collaborative, multicentric and descriptive approach involving 25 institutions from 9 countries is presented. Demographics, clinical manifestations, expanded disability scale status (EDSS), brain and spinal cord MRI, serological anti-AQP4-IgG and anti-MOG-IgG antibodies, and cerebrospinal fluid (CSF) oligoclonal bands were included. A central serological repository utilized the cell-based assay. The specimens outside of this network employed diverse methodologies. Data were collected at the Gorgas Commemorative Institute of Health Studies (ICGES), Panama, and included 186 subjects, of which 84% were females (sex ratio of 5.6:1). Mestizos constituted 72% of the study group. The median age was 42.5 years (IQR: 32.0–52.0). Associated autoimmune diseases (8.1%) were myasthenia gravis, Sjögren’s syndrome and systemic lupus erythematosus. The most common manifestation was optic neuritis-transverse myelitis (42.5%). A relapsing course was described in 72.3% of cases. EDSS scores of 0–3.5 were reported in 57.2% of cases and higher than 7.0 in 14.5%. Positive anti-AQP4-IgG antibody occurred in 59.8% and anti-MOG-IgG antibody in 11.5% of individuals. Antibody testing was lacking for 13.4% of patients. The estimated crude prevalence of NMOSD from Panama and the Dominican Republic was 1.62/100,000 (incidence of 0.08–0.41) and 0.73/100,000 (incidence 0.02–0.14), respectively. This multinational study contributes additional insights and data on the understanding of NMOSD in this Latin American region.

## 1. Introduction

Neuromyelitis Optica Spectrum Disorder (NMOSD) is an inflammatory, demyelinating neurological disorder. NMOSD is pathologically characterized by autoimmune damage of aquaporin channels and astrocytes in the central nervous system (CNS). This results in severe neurological dysfunctions manifesting primordially with optic nerve and spinal cord inflammatory attacks [1]. The disease process may also affect diverse areas of the brain, the diencephalon and, distinctly, the area postrema. In addition, it may produce hypothalamic/pituitary axis alterations with neuro-endocrine manifestations and is commonly associated to serological or clinical expressions of other autoimmune disorders, including myasthenia gravis, lupus erythematosus, Sjögren’s and anti-phospholipid antibody syndromes [2]. The majority of cases have a relapsing course, although monophasic disease is also present. Historically, NMOSD was considered as a severe manifestation of multiple sclerosis (MS) until the 1990s when, due to the identification of specific clinical and magnetic resonance imaging (MRI) characteristics, it became evident that these entities were separate pathologies [3].

Aquaporin-4 (AQP4), a CNS water channel abundantly expressed in astrocytic processes resting at the blood-brain barrier, becomes the target of an IgG antibody in NMOSD, resulting in widespread astrocytic damage in brain and spinal cord. Anti-AQP4-IgG-antibody is exclusively present in the majority of people affected by this disease (60–70%)). Anti-AQP4-IgG-antibody was discovered in 2004 [4], establishing a fundamental differentiation with MS, the prime CNS demyelinating disease, and became the cardinal biomarker for NMOSD. Diverse laboratory techniques may detect the antibody in serum. However, the internationally recommended methodology is the cell-based assay (CBA), which has a sensitivity as high as 92% [5]. A small portion of seronegative patients may test positive for an IgG antibody against myelin oligodendrocyte (MOG). Whereas detailed clinical assessments may differentiate these disorders, characterization studies are currently ongoing for this yet more rare condition: anti-MOG antibody disease.

NMOSD affects women disproportionally in ranges reported from 3:1 to 6:1 [6]. In previous Latin American studies, between 80% and 82.7% of patients were women [7,8]. At the global level, the median age of the onset of disease is 32–40 years, with a slightly higher median age of clinical debut in some Latin American cohorts, reportedly as high as 43.3 years [9]. The most distinct finding by MRI is longitudinally extensive transverse myelitis (LETM), lesions occupying three or more spine levels. The cord lesions may be associated to normal brain images, or to T2/FLAIR abnormalities in one or both optic nerves extending to the chiasm. T2/FLAIR abnormalities may also occur in other regions of the brain rich in AQP4 channels, such as the periependymal layers surrounding the third ventricle and cerebral aqueduct, brain stem, thalamus, hypothalamus, basal ganglia, and subcortical white matter [10]. In general, the brain and spinal cord MRI abnormalities in NMOSD do not conform to the expected pattern for imaging criteria for MS [11].

NMOSD is a relatively rare disease with a low prevalence worldwide, with frequencies in Latin America reportedly ranging between 0.37 and 4.52/100,000 [12]. Even though a great racial and ethnic heterogeneity exists in Latin America, Mestizos, the blended genetics and cultures over the course of five centuries of white Caucasians of European ancestry with Native Americans and black Africans have emerged as the modern predominant Latin American ethnic population. Whereas MS appears to have an increasing prevalence among Latin Americans, NMOSD observations indicate that this disease is also widely identified throughout the continent [13]. The current paper reports a multinational regional collaborative study on the clinical characterization of NMOSD from the six Central American and three Caribbean countries. We feel this collaborative effort contributes additional insights and data to the understanding of this disease in this Latin American region.

## 2. Methods and Study Design

### 2.1. Study Group

A multinational, multicentric, descriptive and ambispective study was designed introducing data on patients diagnosed with NMOSD from January 2010 to December 2020. Only individuals 18 years of age or older were included. Data contributed by 37 certified neurologists and epidemiologists comprising 25 institutions from Guatemala, El Salvador, Honduras, Nicaragua, Costa Rica Panama, Cuba, Santo Domingo and Aruba were collected and analyzed between February and August 2021. Contributing health centers from Costa Rica included Hospital San Juan de Dios CCSS and Hospital San Rafael de Alajuela CCSS, both part of the national health care system. The conglomerate of participating centers constituted institutions from public health and private care, or from social security systems. The cases were identified according to the Wingerchuk et al. revised diagnostic criteria from 2006 [14], and the International Panel for NMOSD Diagnosis, 2015 [15].

### 2.2. Data Collection

A questionnaire designed in a digital format included demographic variables (age, gender, country of birth, race and ethnicity), family history of NMOSD, history of NMOSD (date of onset, date of diagnosis and clinical course) and specific clinical manifestations: optic neuritis, acute myelitis, acute area postrema, diencephalic and brain stem syndromes, and symptomatic cerebral manifestations. The clinical course, monophasic or relapsing, was established at the time of inclusion. Autoimmune comorbidities were also part of the clinical inquiry. Expanded Disability Scale Status (EDSS) measurements; findings of brain, cervical and thoracic MRI studies; laboratorial determinations of serological anti-AQP4-IgG and anti-MOG-IgG; and cerebrospinal fluid (CSF) oligoclonal bands completed the purposive sampling. Data were centrally collected at the Gorgas Commemorative Institute of Health Studies (Spanish abbreviation: ICGES) in Panama City, Panama. Three Google applications were employed: Drive = https://drive.google.com/drive/, Forms = https://docs.google.com/forms/, and Sheet of calculus = docs.google.com/spreadsheets/ (accessed on 15 September 2021), to elaborate the instrument of data concentration according to the questions established by the protocol. This structure was shared through electronic correspondence with the lead investigator from each country, and data were stored in real-time in a Google calculus sheet for further implementation. Each investigator received a manual detailing the methodology for capturing information. To guarantee adequate data acquisition, a pilot trial was performed prior to the formal initiation of the study.

Investigators were able to readily access their data. The collected information on the electronic server of ICGES may eventually be utilized for NMOSD national registries or as a regional depository after the participating centers obtain approval from their respective regulatory institutions. An alphanumeric code was assigned to each patient to protect his or her identity, and their personal information was eliminated once the absence of duplication was assured through an external audit.

### 2.3. Serological Sample Acquisition

Laboratory technology to detect anti-AQP4-IgG antibody, particularly CBA technology, the most sensitive technique, are not available in the region. Some serum samples were processed in laboratory facilities outside the country of the investigator. For this study, a central laboratory was employed. Following a signed consent, 10 cc of peripheral blood were drawn, and via local certified private laboratories from each country, shipped to the ECHANDI Laboratory in San Jose, Costa Rica, serving as central processing site for determination of anti-AQP4-IgG antibody, utilizing CBA methodology. In case of a negative result, the specimen was assessed for anti-MOG-IgG antibody. Once the process was completed, the remnant sample was discarded. Utilization of the ECHANDI laboratory assisted participating investigators who had no access to determination of anti-AQP4-IgG antibody technology. Nevertheless, the protocol allowed the inclusion of subjects who underwent testing utilizing other serological methodologies, such as Immunoprecipitation and enzyme-linked immunosorbent assay (ELISA), and tissue-based indirect immunofluorescence (IIF). The determination of oligoclonal bands (OCBs) in cerebrospinal fluid (CSF) was included in the protocol to assist in the differential diagnosis with the main demyelinating disorder, MS, and in view of the lack of local access to specific serological testing for NMOSD.

### 2.4. Statistical Analysis

All demographic and historical data, comorbidities association, clinical variables, MRI findings and laboratory results were analyzed. Descriptive measures of central tendency (median) and dispersion measurement (IQR = interquartile range) were used to analyze age, timing of diagnosis and age at the presentation of the first symptom. Proportions were utilized for the analysis of categorical variables, and the Chi² test was applied for comparisons. The prevalence rate, with a confidence interval (CI) of 95%, was estimated by the number of living patients with NMOSD at age 18 or older, as the numerator among the adult population (18 years and older) and as the denominator per 100,000 inhabitants. Crude prevalence per country, per gender and per age group were estimated according to the NMOSD population determined on 1 July 2020. The incidence rate, with a CI of 95%, was calculated from the number of NMOSD cases included from 1 January 201, to 31 December 2019 as the numerator, and the complete estimated population number (1 July 2019) of patients-years at risk as the denominator per 100,000 inhabitants. Any variable with a value of *p* < 0.05 was considered statistically significant. Epi Info™ (Build 7.2.4—27 April 2020) was used to perform the statistical analysis.

### 2.5. Ethical Considerations

Each investigator contributed data from their institutional or private practice files. Considering that patients’ private information was not utilized, and their identities were protected, an informed consent waiver to use these data was obtained from the respective Institutional Review Boards (IRBs) or Ethics Committees for all participants, except for patients who required blood drawing for the disease biomarkers process. The study was conducted according to the guidelines of the Declaration of Helsinki, and the criteria established by the U.S. Department of Health and Human Services, 45 Code of Federal Regulation for Protection of Human Subjects, part 46, subpart A. IRB approval was obtained from all participating institutions.

## 3. Results

The screening phase of the study included 229 patients, of which 39 candidates were excluded because their onset of disease was outside the inclusion study epochs, and another 4 were of pediatric age. The final sample constituted 186 subjects, of which 84% (158) were female, providing a sex ratio of 5.6: 1. The majority of patients, 72.0% (*n* = 134), were identified as Mestizos (Table 1). This group included biracial individuals derived from white Caucasian and Native American fusions, or white with black of African ancestry origin, the typical ethnic/racial expressions in Latin America. The largest proportion of white Caucasians (80.0%; *n* = 10) was reported from Cuba. There were 11 black Afro descendants reported from Panama (*n* = 5), Cuba (*n* = 3), Aruba (*n* = 2) and the Dominican Republic (*n* = 1). One individual from Panama was identified as Native American.

The median age was 42.5 years (IQR: 32.0–52.0), while the median age at the onset of disease was 37.0 years (IQR: 28.0–48.0). The median time of the duration of disease was 6.8 moths (IQR: 1.0–34.5). Aruba, Costa Rica, El Salvador, Honduras and Panama reported a median time between 1.2 and 6.3 months to accomplish the diagnosis. Longer times were reported from Cuba (60.9 months), Guatemala (18.7 months) and Nicaragua (16.4 months). The shortest time was reported from Aruba (1.2 months) (Table 2).

The presence of autoimmune disorders was reported in 8.1% (15/186) of cases. The most common (*n* = 7) was myasthenia gravis, followed by Sjögren’s syndrome (*n* = 3) and systemic lupus erythematosus (*n* = 2). The most common clinical manifestation was the association of optic neuritis (ON)-transverse myelitis (TM) in 42.5% of cases. Only TM was present in 25.3%, and only ON was present in 16.7%. Other frequent combinations of clinical symptoms were reported in 26 patients (13.97%) as ON, TM and area postrema syndrome (Table 3). EDSS determinations were performed in 152 patients. Disability scores of 0–3.5 was reported in 57.2% (*n* = 87) of patients, while 28% (*n* = 43) showed scores of 4.0–6.5, and 14.5% (*n* = 22) showed scores higher than 7.0. The clinical course was relapsing in 72.3% of cases.

MRI studies showed abnormalities compatible with NMOSD in the cervical cord (68.9%; *n* = 124/180), thoracic cord (67.1%; *n* = 110/164) and brain (51.2%; *n* = 88/173). Every patient underwent at least one MRI study, and each study was abnormal, contributing to the diagnosis of NMOSD by adhering to the described imaging characteristics of the disease. Anti-AQP4 antibody serological studies in 164 patients resulted in 59.8% (98 patients) positivity. Anti-MOG antibody testing in 87 patients was positive in 11.5% (10/87), with all subjects having tested negative for anti-AQP4 antibody assays (Table 4). From this group, 32 patients were negative for both assays. Samples processed in laboratory facilities outside the investigator’s country (not using the centralized repository) were tested with immunofluorescence and ELISA techniques in 21.35% (*n* = 34) and 4.7% (*n* = 4) of these cases with AQP4 and anti-MOG, respectively. Utilizing IIF methodology, 15.09% (*n* = 24) of samples tested anti-AQP4-IgG-positive, and 11.64% (*n* = 10) tested anti-MOG-IgG antibody-positive. Antibody testing was not performed on 13.44% of cases of patients clinically diagnosed with NMOSD, while 53.8% (100/186) did not undergo anti-MOG-IgG antibody assessments.

Considering that serological testing was not readily available in the region, to establish a more accurate differential diagnosis, OCBs were determined in CSF in some cases. This abnormality was detected in 40% of cases (100/186). Nevertheless, the performance of this study was not consistent in the participating countries: none in Cuba, 1/7 in El Salvador, 1/6 in Nicaragua, 4/7 in Honduras and 4/4 in Aruba. None of the OCB determinations from these countries showed positive findings. OCBs were present, however, in 65% (13/27) of patients from Costa Rica, 52.2% (46/51) from the Dominican Republic, and 40% (8/20) from Panama (Table 4).

The national prevalence and incidence data from two countries, Panama and the Dominican Republic, could be extracted from the information provided from their total contributing centers and institutions. Data from the rest of countries of the region were cross-sectional, institutional results. The crude prevalence of NMOSD in Panama and the Dominican Republic was estimated as 1.62 per 100,000 inhabitants and 0.73 per 100,000 inhabitants, respectively. The female rate prevalence in Panama was 2.62, while male rate was 0.61/100^3^ inhabitants (*p* < 0.0001). In the Dominican Republic, the female rate was 1.19, and the male rate was 0.26/100^3^ inhabitants (*p* < 0.0001). The prevalence increased with age (30–50 years) in both countries and tended to decline after 60 years of age. NMOSD incidence varied between these two countries: 0.08–0.41 per 100,000 inhabitants in Panama, and 0.02–0.14 in the Dominican Republic. Since 2015, the diagnoses of NMOSD have increased in these countries.

## 4. Discussion

At the time when NMOSD was stated to be clinically separated from MS in the 1990s, the initial observations in the Central American-Caribbean (CA-C) region appeared from the French West Indies, namely from Guadeloupe and Martinique [16,17]. Studies from Cuba [18] and Brazil [19] further contributed to the notion that this disorder appears to involve population groups commonly not affected by MS. Despite its complex ethnic composition across the subcontinent, Latin America shows a remarkable societal, cultural and linguistic interrelationship. The current study addressing NMOSD characteristics from the six Central American and three Caribbean countries, all Spanish-speaking nations except Aruba, showed common ethnic distributions. The study showed the majority of the cases (72.0%) were Mestizos, the predominant and typical ethnic expression of people in this part of the world, and confirmed the preponderance of the disease in women (female/male ratio: 5.6:1). These findings are not discordant from other Latin American studies [20]. In these CA-C series, NMOSD presented clinically with a median age of 37.0, eventually initiating a relapsing course in reportedly 72.3% of patients. The median age of onset for the CA-C cohorts is 6.3 years younger than the median age of onset reported from other Latin American studies [21].

The common involvement of the spinal cord as transverse myelitis (a core manifestation of the disease) was ascertained by MR imaging in 68.9% of cervical and 67.1% of thoracic studies. This phenotypic tendency was reflected in 28% of cases exhibiting a high EDSS (4.0–6.5), thus indicating impaired ambulation and the need for an assisted walking device. Moreover, 14.5% of patients showed a total inability to walk and confinement to a wheelchair (EDSS > 7.0). These findings indicate the aggressive clinical behavior of this disease. Brain MRI abnormalities were present in 51.2% of cases. The prolonged times to reach diagnosis exhibited by some countries suggests local problematic health care efficiencies and access to adequate diagnostic tools, common barriers faced in most Latin American countries.

Serological assays showed a positive antibody anti-AQP4 in 59.8% of cases, while 11.5% were anti-MOG antibody-positive (using CBA technology). The use of other laboratory methodologies employed in some of these cases were allowed by the protocol. The degree of sensitivity of these assays is lower than CBA, but still acceptable. These other technologies are less expensive and widely used in the region. Despite the testing facilities offered by the protocol, 13.4% (22/186) of patients did not undergo a serological study.

Given the lack of serological testing for NMOSD in the CA-C region, in some cases, the determination of OCBs in CSF was integrated into this protocol to facilitate differential diagnoses with MS. Although OCBs were reported in patients from Costa Rica, the Dominican Republic and Panama cohorts, this finding was inconsistent and appeared in the context of patients clinically and serologically established as NMOSD.

This CA-C NMOSD study provides not only the first clinical characterization from the region but also sets the basis for national and regional registries.

## 5. Limitations

There were several limitations in this study. Complete national data on NMOSD from the individual CA-C countries remain under study. Not all of these countries have the institutional capabilities to provide coordinated national data. Obtaining the appropriate specific serological tests for NMOSD in CA-C emphasizes the importance and unmet need of having access to specialized laboratory investigations for this disease and related disorders in this region. Therapeutic management was not addressed in this work since a recent study surveying all countries from Latin America, including the region here reported [21], showed the widespread and sole utilization of symptomatic and off-label therapies, with the advent of recently licensed drugs just beginning to emerge in these countries.

## 6. Conclusions

NMOSD remains a low-prevalence, low-incidence neurological disease around the globe, albeit carrying a great societal impact due to the degree of disability exerted in the affected population groups. Studying NMOSD in the different regions of Latin American offers a unique opportunity to assess the epidemiology, clinical behaviors and general aspects of this disease. This is the first study providing information and data on the clinical characterizations of NMOSD from nine countries and multiple cohorts from CA-C.

## Figures and Tables

**Table 1:** Demographic characteristic of NMOSD patients from Central America and the Caribbean.

País.	Number	Female (%)	Mestizo Ethnicity (%)
Aruba	4	100.0	50.0
Costa Rica	27	85.2	33.3 *
Cuba	13	84.6	0.0 ^a^
El Salvador	7	100.0	85.7
Guatemala	23	82.6	100.0
Honduras	7	100.0	85.7
Nicaragua	6	100.0	100.0
Panama	48	81.3	79.2
Dominican Republic	51	82.4	86.3
Total	186	84.9	72.0

* 22.2% Caucasian and 44.4% no data. ^a^ 80.0% Caucasian.

**Table 2:** Age, median age of onset, time to diagnosis and interquartile range.

Country	Number	Median Age	Median Age IQR *	Median Age at Onset	Median Age at Onset IQR	Median Age at Diagnosis	Median Age at Diagnosis IQR	Median Time to Diagnosis (Month)	Median Time to Diagnosis IQR
Aruba	4	32.5	24.5–44.0	30.0	20.0–41.5	30.0	20.0–41.5	1.2	1.0–2.7
Costa Rica	27	47.0	38.0–56.0	44.0	32.0–52.0	45.0	33.0–54.0	6.1	1.0–21.3
Cuba	13	29.0	21.0–47.0	24.0	14.0–36.0	28.0	19.0–41.0	60.9	48.7–60.9
El Salvador	7	44.0	36.0–54.0	37.0	34.0–44.0	39.0	35.0–44.0	6.3	1.6–16.9
Guatemala	23	44.0	37.0–48.0	36.0	29.0–46.0	39.0	31.0–47.0	18.7	6.2–50.3
Honduras	7	35.0	31.0–45.0	33.0	25.0–38.0	33.0	28.0–42.0	2.4	1.4–43.9
Nicaragua	6	47.5	44.0–54.0	44.5	38.0–46.0	45.0	38.0–53.0	16.4	10.6–35.5
Panama	48	42.5	31.5–51.0	37.5	28.5–49.0	38.5	29.0–50.0	1.5	0.1–12.2
Dominican Republic	51	41.0	34.0–54.0	36.0	30.0–48.0	39.0	30.0–52.0	7.7	2.0–46.7
Total	186	42.5	32.0–52.0	37.0	28.0–48.0	39.0	30.0–50.0	6.8	1.0–34.5

* IQR = Interquartile range (25–75%).

**Table 3:** Relative frequency of NMOSD clinical symptoms in Central America and the Caribbean (186).

Clinical Symptoms	Frequency	Percentage (%)
Optic Neuritis-Transverse Myelitis	79	42.5
Transverse Myelitis	47	25.3
Optic Neuritis	31	16.7
Brain Stem Syndrome	1	0.5
Cerebral Syndrome	1	0.5
Area Postrema Syndrome	1	0.5
Syndromic Combinations	26	14.0

**Table 4:** Profile of serum antibodies and oligoclonal bands (CSF) in 186 patients with NMOSD in Central America and the Caribbean.

Country	AQP4-IgG Ab	Positivity (%)	MOG-IgG Ab	Positivity (%)	OCB	Positivity (%)
Aruba	3	66.7	2	50.0	4	0.0
Costa Rica	24	75.0	7	14.3	13	61.5
Cuba	11	72.7	-	-	-	-
El Salvador	7	71.4	-	-	1	0.0
Guatemala	21	66.7	12	8.3	11	0.0
Honduras	5	100.0	4	0.0	4	0.0
Nicaragua	5	60.0	3	0.0	1	0.0
Panama	38	50.0	13	23.1	20	40.0
Dominican Republic	50	48.0	46	8.7	46	52.2
Total	164	59.8	87	11.5	100	40.0

Ab: Antibodies; OCB: Oligoclonal Bands.

## Data Availability

The data presented in this study may be available on reasonable, approved request from the corresponding author.
